# Experimental rat model for acute tubular injury induced by high water hardness and high water fluoride: efficacy of primary preventive intervention by distilled water administration

**DOI:** 10.1186/s12882-020-01763-3

**Published:** 2020-03-24

**Authors:** Thanusha Perera, Shirani Ranasinghe, Neil Alles, Roshitha Waduge

**Affiliations:** 1 Postgraduate Institute of Science, University of Peradeniya, Peradeniya, Sri Lanka; 2 Department of Biochemistry, Faculty of Medicine, University of Peradeniya, Peradeniya, Sri Lanka; 3 Department of Pathology, Faculty of Medicine, University of Peradeniya, Peradeniya, Sri Lanka

**Keywords:** Chronic kidney disease of unknown etiology (CKDu), Water hardness, Drinking water, Acute tubular injury, Serum creatinine, Serum urea, Aspartate aminotransferase (AST) activity

## Abstract

**Background:**

High water hardness associated with high water fluoride and the geographical distribution of Chronic Kidney Disease of unknown etiology (CKDu) in Sri Lanka are well correlated. We undertook this study to observe the effects of high water hardness with high fluoride on kidney and liver in rats and efficacy of distilled water in reducing the effects.

**Methods:**

Test water sample with high water hardness and high fluoride was collected from Mihinthale region and normal water samples were collected from Kandy region. Twenty-four rats were randomly divided into 8 groups and water samples were introduced as follows as daily water supply. Four groups received normal water for 60 (N1) and 90 (N2) days and test water for 60 (T1) and 90 (T2) days. Other four groups received normal (N3) and test (T3) water for 60 days and followed by distilled water for additional 60 days and normal (N4) and test (T4) water for 90 days followed by distilled water for another 90 days. The rats were sacrificed following treatment. Serum samples were subjected to biochemical tests; serum creatinine, urea, aspartate aminotransferase (AST), alanine aminotransferase (ALT), alkaline phosphatase (ALP) and elemental analysis. Histopathological examinations were carried out using kidney and liver samples.

**Results:**

Test water treated groups were associated with acute tubular injury with loss of brush border and test water followed with distilled water treated groups maintained a better morphology with minimal loss of brush border. Serum creatinine levels in T1 and T2 groups and urea level in T2 group were significantly (*p* < 0.05) increased compared to control groups. After administration of distilled water, both parameters were significantly reduced in T4 group (*p* < 0.05) compared to T2. Serum AST activity was increased in T4 group (*p* < 0.05) compared to control group with no histopathological changes in liver tissues. The serum sodium levels were found to be much higher compared to the other electrolytes in test groups.

**Conclusion:**

Hard water with high fluoride content resulted in acute tubular injury with a significant increase in serum levels of creatinine, urea and AST activity. These alterations were minimized by administering distilled water.

## Background

Chronic kidney disease (CKD) is viewed as part of the rising worldwide non-communicable disease burden. Hypertension and diabetes mellitus are the important risk factors for this disease in all developed and many developing countries [1]. In early nineties, there has been a rising incidence and prevalence of chronic renal failures that has emerged in the North Central region (NCR) of Sri Lanka where the disease is not associated with any known risk factors [2]. Due to the elusive nature of the disease, it has been named “Chronic Kidney Disease of unknown etiology” (CKDu). Because of its slow progressive loss of kidney function, CKDu often gets worst slowly and remain undiagnosed over a long period of time [3].

Ground water source is considered as a causative factor for CKDu [4, 5]. The elevated levels of fluoride, which is defined as above 0.5 mg/L by the World Health Organization in groundwater sources is observed in CKDu endemic regions [6]. Therefore, fluoride has received increased attention as a risk factor in the etiology of CKDu [7, 8]. Jayasumana et al (2014) and other groups reported that water hardness could contribute to the etiology of CKDu due to exceeding the levels of ideal water hardness of between 150 and 250 mg/L CaCO_3_ [9–11]. Moreover, the hydrogeochemical investigations in CKDu endemic areas revealed that both fluoride and hardness is elevated in all CKDu endemic regions [12].

**Fig. 1:**
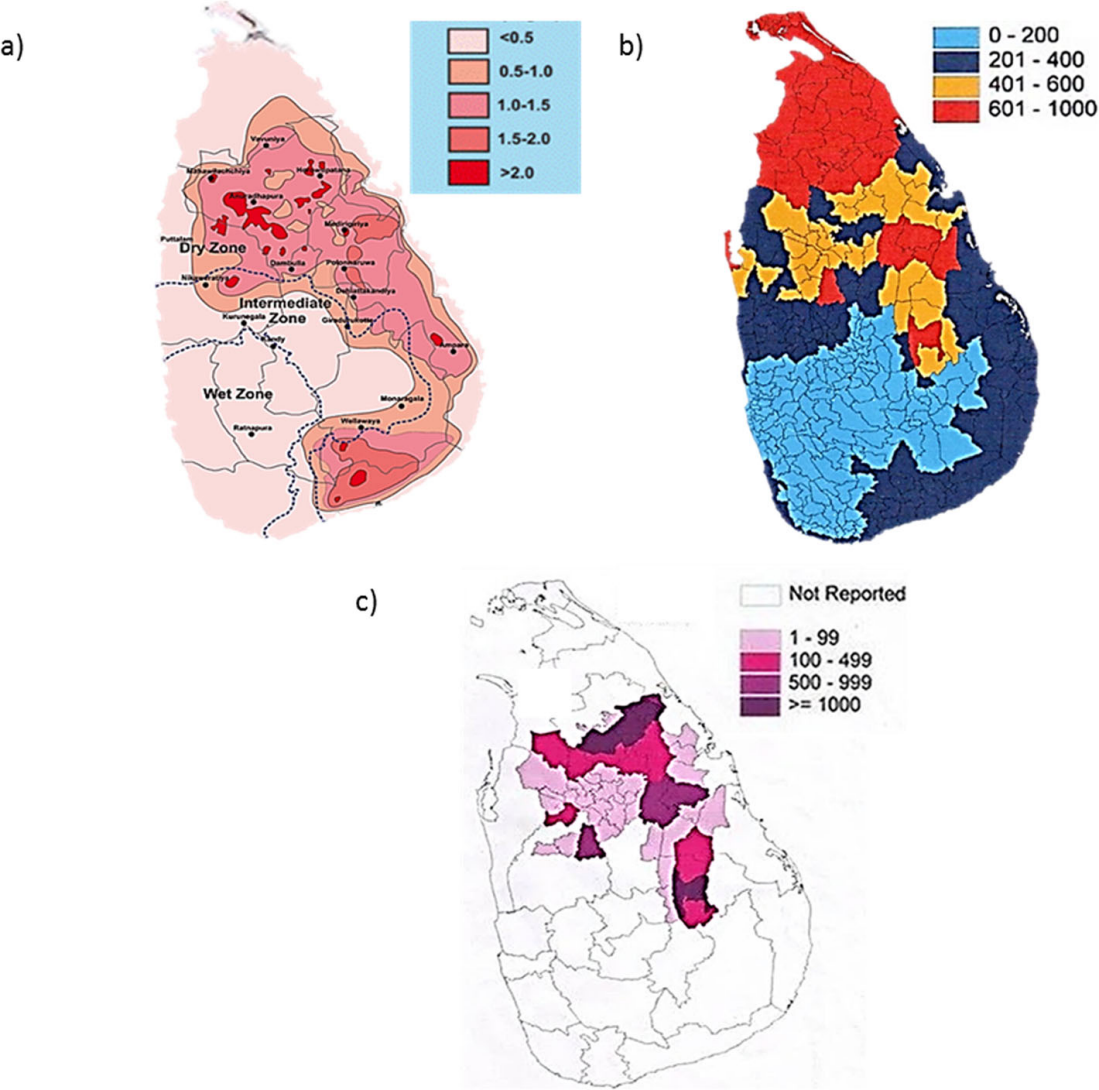
Geographical distribution of water hardness, ground water fluoride and CKDu patients in Sri Lanka. **a** Distribution of fluoride in ground water in Sri Lanka (Source: Chandrajith et al.; 2012) **b** Ground water hardness variation over Sri Lanka (Source: National water supply and drainage board, Sri Lanka; www.waterboard.lk) **c** Distribution and prevalence of CKDu in Sri Lanka (Source: Jayasumana et al.; 2014)

Previous studies revealed that high concentrations of groundwater fluoride distributed in the dry zone, whereas minimal fluoride levels were reported in the wet zone (Fig. 1a). Further, Chandrajith et al. (2011), reported high fluoride levels from 1.3–5.3 ppm in CKDu endemic areas such as Girandurukotte, Nikawewa, Madawachchiya and Padaviya in Sri Lanka [8]. These findings were further confirmed by the occurrence of clinically diagnosed dental and skeletal fluorosis in these areas [13, 14]. In contrast, CKDu occurrence was less even in the areas with high concentrations of groundwater fluoride such as Huruluwewa and Wellawaya [8].

The hardness of drinking water is mainly dependent on the concentration of dissolved cations namely, calcium and magnesium predominantly in combination with the anions, bicarbonate, sulphate and chloride [15]. Other cations such as Al, Zn, Ba, Fe, Sr, and Mn have minor contribution for the total hardness of water [16, 17]. Increased total water hardness is common in northern and northern central parts of Sri Lanka (Fig. 1b). Jayasumana et al (2014), reported high water hardness (up to 820 mg/L) in CKDu endemic areas which showed a correlation with the prevalence of CKDu [3, 18]. But, unexpected low occurrence of CKDu was found in Jaffna and Puttalam districts even with reported higher water hardness (approximately 1500 mg/L) [19, 20].

When we overlapped the high fluoride areas with high water hardness areas surprisingly, the same areas were overlapped with the reported high occurrences of CKDu namely Medawachchiya, Girandurukotte, Kabithigollawa, Padaviya, Medirigiriya, Dehiattakandiya and Nikawewa regions in the dry zone of Sri Lanka (Fig. 1c) [21].

Therefore, we hypothesized that both high fluoride and high water hardness together contributes to the pathogenesis of CKDu. In our previous study, the effect of fluoride alone on kidney and liver were investigated and proved that there is a possibility of inducing renal damage by elevated serum creatinine levels with exposure to extremely high fluoride levels (20ppmF) for longer period but not with low concentrations [22]. In this study, we performed a comparative study of the effect of both high fluoride and high water hardness on kidney and liver in rats by treating orally a water sample collected from CKDu endemic area. And also, we examined whether the distilled water could reverse the damage created by these factors.

## Methods

Water samples: A water sample with high fluoride and high hardness was collected in June 2017 from a selected dug well in Mihinthale area, a region located in North Central Province of Sri Lanka (8 20′ 57″N 80 30′ 03″E) as a test water sample based on the regional water quality information gathered from the Department of Geology, University of Peradeniya, Sri Lanka. Normal water sample was collected from Kandy, a region located in Central province of Sri Lanka (7.2582° N, 80.5988° E), as the control water sample. Prior to sampling, all the sampling containers were soaked in 10% HNO_3_ for 24 h and rinsed thoroughly with distilled water.

### Analysis of physical and chemical parameters of water

The water samples were analyzed for the physio-chemical parameters such as pH, electrical conductivity (EC), Total dissolved solids (TDS), hardness, alkalinity, fluoride and other common elements namely, Potassium (K), Sodium (Na), Calcium (Ca), Magnesium (Mg), Copper (Cu), Strontium (Sr), Manganese (Mn), Zinc (Zn), Nickel (Ni), Lithium (Li) and Ferrous (Fe) at the department of Geology, University of Peradeniya, Sri Lanka. The pH was measured using a Thermo Orion 290A+ digital pH meter (Thermo Fisher Scientific, Waltham, USA). EC and TDS were determined using a digital Hach Sension5 meter (Hach, Colorado, USA) and total hardness and alkalinity were measured using EDTA and Sulfuric acid titrations, respectively. The fluoride concentrations were measured using a Thermo Scientific Orion fluoride ion selective electrode (Thermo Fisher Scientific, Waltham, USA) and K, Na, Ca, Mg, Cu, Strontium Sr, Mn, Zn, Ni, Li and Fe were analyzed using Thermo Scientific iCE 3000 series Atomic Absorption Spectrophotometry (Thermo Fisher Scientific, Waltham, USA). The results were then matched with WHO standards [23].

#### Animals

Four weeks old 24 male Wistar rats (origin; Clea Japan, Inc.) weighing (200 ± 10 g) were purchased from Medical Research Institute, Sri Lanka and were housed in a polycarbonate cages at 25 °C and 50% humidity on a 12-h light/dark cycle. They were acclimated for 1 week with food and water ad libitum before starting the experiment. The experimental procedures were reviewed and approved by the Ethics Committee of Postgraduate Institute of Science, University of Peradeniya, Sri Lanka and the animal treatment and handling were carried out according to the International Guiding Principles for Biomedical Research Involving Animals (Council for the International Organizations of Medical Sciences 2012) [24]. Each rat was given a unique number and their initial body weights were recorded.

#### Experimental protocols

**Fig. 2:**
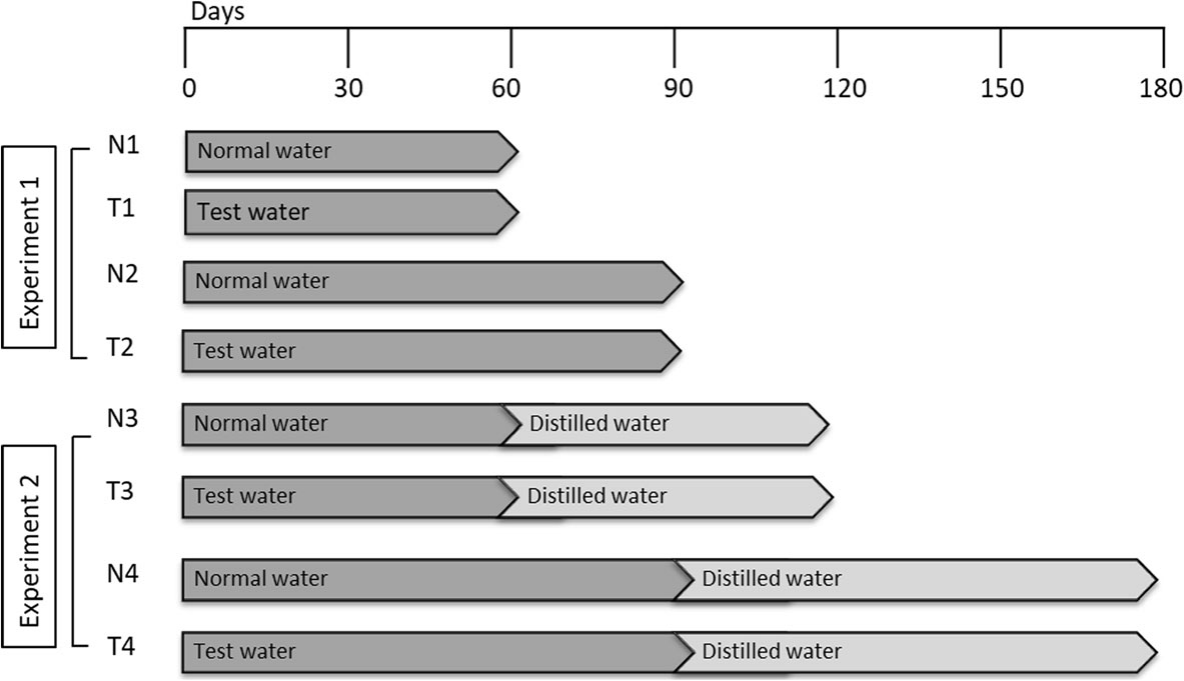
Schematic representation of the experimental design

This study was consisted of two protocols. The first protocol was conducted to assess the effects of fluoride and water hardness on renal and liver parameters of rats (Experiment I). And in the second protocol (Experiment II), reversibility of these effects and the effects of distilled water treatment were evaluated and compared (Fig. 2).

According to Charan (2013) and Arifin (2017), we have calculated the minimal number of animals before beginning the experiment to maintain the scientific quality of the experiment and the relevant results to avoid statistical confusions and ethical issues [25, 26]. After 1 week of adaptation, animals were randomly divided into 8 groups, each with 3 rats and water samples were introduced as daily water supply: The N1 and T1 groups received normal and test water for 60 days respectively and N2 and T2 groups received normal and test water for 90 days. N3 and T3 groups received normal and test water for 60 days and followed with distilled water for additional 60 days. Similarly, the N4 and T4 groups received normal and test water for 90 days and followed with distilled water for another 90 days.

At the end of each treatment period, body weight of each rat was recorded as final body weight and anaesthetized by CO2 inhalation (30% of the cage volume per minute) in an empty chamber until complete cessation of breathing and movement were observed for a minimum of 3 min. Then the rat was placed in ventral decumbency, an incision was made parallel to the midline and cut across abdominal cavity, diaphragm and upper chest. Blood from each animal was withdrawn from their hearts and transferred to polyethylene tubes of each sacrificed rat. Liver and kidneys were dissected out and their fresh weights were recorded.

### Determination of body weight gain and relative organ weights

The body weight gain of each rat was assessed by the difference between final body weight; on the day of sacrifice and initial body weight; before the commencement of dosing. The comparison of the organ weights of treated animals with untreated animals is often complicated by differences in body weights between groups. Therefore, the ratios of the organ weight to body weight were calculated to account for differences in body weights as follow:

$$ \mathrm{Relative}\ \mathrm{organ}\ \mathrm{weight}=\frac{\mathrm{Fresh}\ \mathrm{organ}\ \mathrm{weight}\ \left(\mathrm{g}\right)\times 100}{\mathrm{Body}\ \mathrm{weight}\ \left(\mathrm{g}\right)} $$

### Sample collection

Serum was separated from the blood following centrifugation at 5000 rpm for 5 min and serum was stored at − 20^0^ C until use for the analysis of biochemical parameters and heavy metals. Liver and kidney samples were taken for the histopathological scoring.

### Histological examination

Freshly dissected kidney and liver samples from each animal were fixed in 10% formalin for 24–48 h and dehydrated in graded concentrations of ethanol and cleared in xylene. The fixed tissues were embedded in paraffin, sectioned at 5 μm thickness, and stained with haematoxylin and eosin (HE) for histological examination under a light microscope [27]. Renal histological damage was quantified by the EGTI (Endothelial, Glomerular, Tubular, Interstitial) scoring system devised specifically for animal research on kidney tissue in the context of injury [28].

### Biochemical assays

Serum samples were subjected to biochemical tests namely, serum creatinine (Agappe, Kerala, India), urea (Agappe, Kerala, India), aspartate aminotransferase (AST, Randox, Crumlin, United Kingdom), alanine aminotransferase (ALT, Agappe, Kerala, India), and alkaline phosphatase (ALP, Fortress, Northern Ireland, United Kingdom). Tests were performed using diagnostic kits and absorbances were measured using a spectrophotometer (Shimadzu, Kyoto, Japan).

### Determination of different element levels in serum

All serum samples were analyzed for K, Na, Ca, Mg, Cu, Sr, Mn, Zn, Ni, Li and Fe using Thermo Scientific iCE 3000 series Atomic absorption spectrophotometer (Waltham, MA, USA). Fluoride levels in serum samples were analyzed using the F-ion selective electrode (Thermo Scientific Orion, USA).

### Statistical analysis

All values were expressed as the mean SD. Data were compared by using one-way analysis of variance (ANOVA) and Fisher’s multiple comparison tests. Values of *P* < 0.05 were accepted as significant.

## Results

### Water analysis

The physio-chemical parameters of ground-water need to be studied to determine the water quality, which provides information about safety and water suitability for drinking purposes. Therefore, normal water and test water samples were subjected to water quality parameters and analyzed as mentioned in the methodology section. Then the obtain results were compared with WHO standards [23] (Table 1).

**Table 1:** Physio-chemical parameters of studied water samples. All concentrations are expressed in mg/l unless otherwise specified

Samples	pH	TDS	Alk	HD	EC (μS/cm)	F	Na^+^	K^+^	Ca^2+^	Mg^2+^
Control water	6.62	47.5	32	84	94.5	0.20	38.22	2.97	5.13	11.13
Test water	9.38^a^	854	284^a^	364^a^	1212	1.66^a^	187.11	6.65	70.00	21.17

TDS-total dissolved solid; Alk-total alkalinity; HD-total hardness; EC-electrical conductivity

^a^Comparatively higher from WHO standard limits

It was noticed that pH, alkalinity, hardness and fluoride were higher from WHO standard limits of 6.5–8.5, 200 mg/L, 250 mg/L and 0.5 mg/L respectively and all the other parameters were within the allowed values. As regards to the major elements, Na^+^ and Ca^2+^ were much higher in test water sample than in tap water sample and much closer to WHO limits of 200 mg/l and 75 mg/l respectively.

The occurrence of trace elements in groundwater is more common in agricultural regions and elevated levels may harmful for human health. Therefore, the water samples were subjected to chemical analysis to determine its quality and all trace elements in both samples were under the permissible limits of WHO standards (Data not shown).

### No differences in weight gain outcome by intervention group

**Fig. 3:**
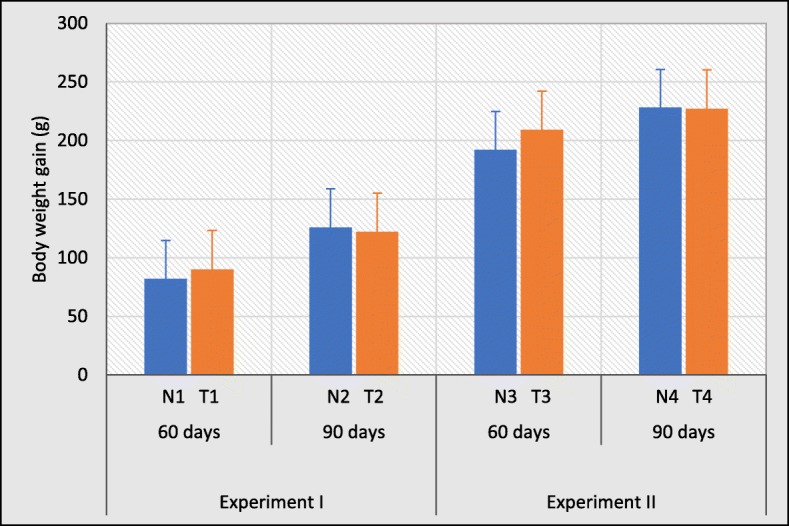
Weight gain distribution of rats treated with normal water and test water ad libitum for 60 and 90 days (Experiment I) and distilled water treated groups as a treatment for 60 and 90 days (Experiment II). Error bars represent standard error. Data are expressed as mean ± S.D., (*n* = 3)

Measures of animal growth are routinely evaluated in toxicology studies and are key to interpretation of compound related effects [29]. Therefore, the body weight gain was calculated as an indicator of toxic effect. According to the results, the body weight gain of the rats in experiment I were not significant between normal water treated groups and test water treated groups for 60 (N1 with T1) and 90 days (N2 with T2). And also, the body weight gain remained largely unaltered between test groups and their respective control groups treated with distilled water for 60 (N3 with T3) and 90 days (N4 with T4) in experiment II (Fig. 3). However, the body weight gain was increased gradually over the experiment for all groups with no significant difference in weight gain among the groups with respect to their control groups. Therefore, the body weight gains did not affect the survival of the rats or cause any gross signs of toxicity.

### No significant changes in the relative weights of kidneys and liver of treated rats in relation to control groups

**Fig. 4:**
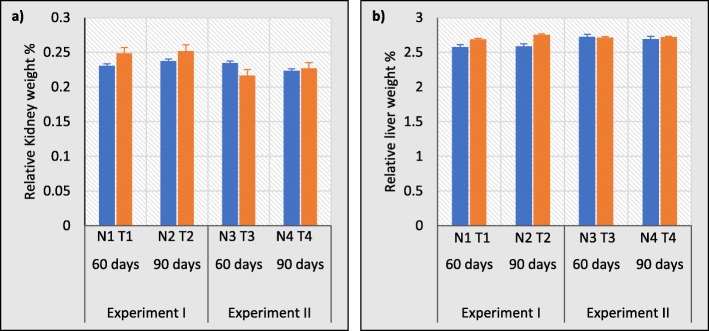
Relative **a** kidney and **b** liver weights of rats exposure to normal water and test water for 60 and 90 days (Experiment I) and distilled water as a treatment for 60 and 90 days (Experiment II). Error bars represent standard error. Data are expressed as mean ± S.D., (n = 3)

Then, the ratio of organ weight to body weight of the rats were evaluated to assess the effects on specific organs as a criterion of response. Therefore, liver and kidneys were analyzed using organ-body weight ratio to evaluate the effects of normal water, test water and distilled water samples on organ weights (Fig. 4). The test groups treated with test water showed a considerable increase in the relative kidney weights after 60 and 90 days in experiment I (T1 and T2) and slight decrease after 60 days (T3) of distilled water treatment in experiment II compared to control groups (Fig. 4a). On the other hand, relative liver weights (Fig. 4b) were also increased slightly after exposure to test water for 60 and 90 days (T1 and T2) but not in experiment II. Although they were much differences in relative organ weights, all these alterations were not statistically significant.

### Test water improves renal histopathological damage following acute tubular injury

**Fig. 5:**
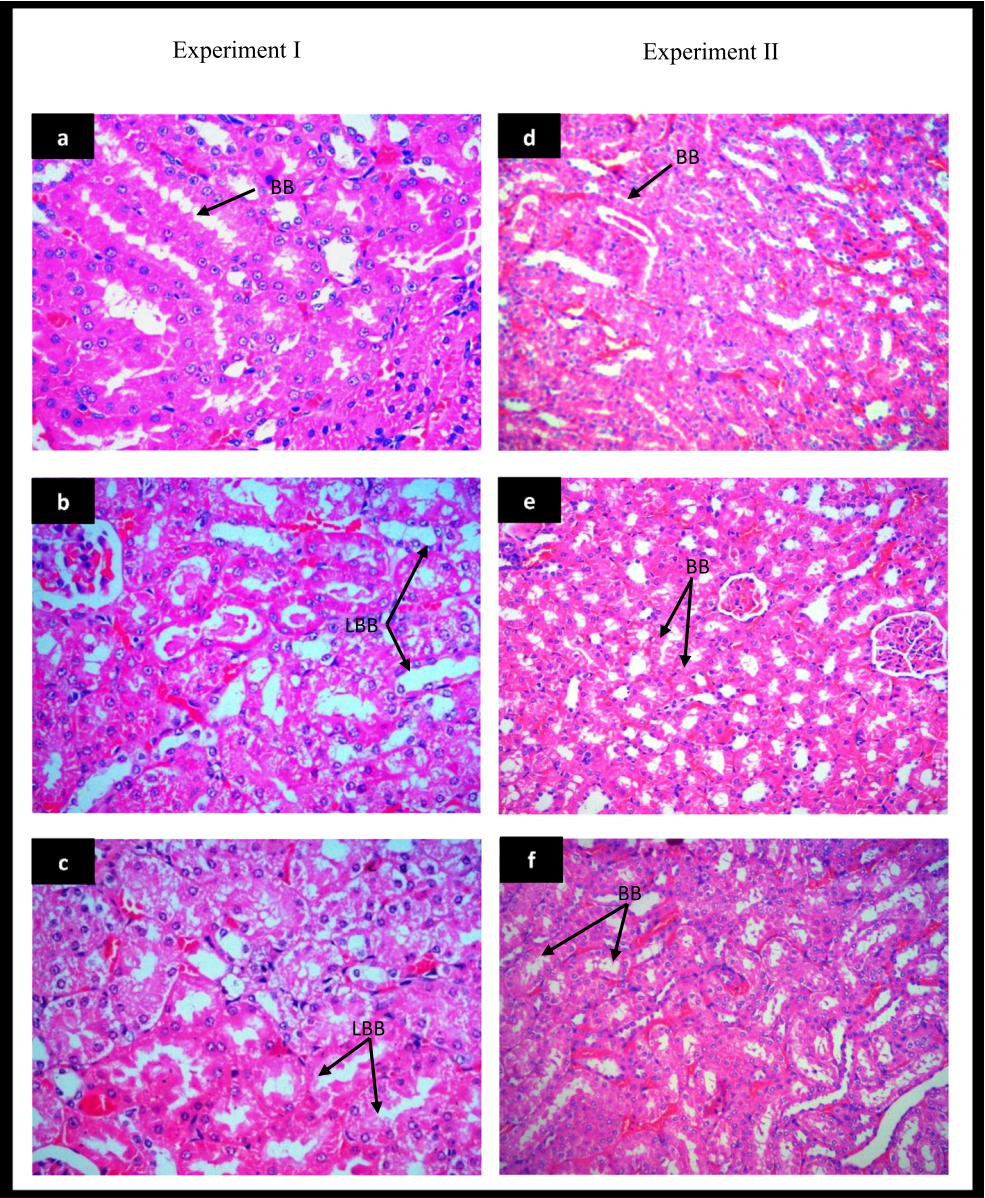
Histopathological examination of kidney tissues stained with hematoxylin-eosin (HE) under light microscope (200X). **a** Control rats showing normal rat kidney with normal tubular brush-borders (BB); **b** tubular brush-borders loss less than 25% of tubular cells and integrity of basal membrane in test water treated rats for 60 days; **c** loss of tubular brush-border in more than 25% of tubular cells and thickened basal membrane in test water treated rats for 90 days; **d** Distilled water treated control rats with normal morphology of kidney; improved tubule architecture in distilled water treated rats for 60 (**e**) and 90 days (**f**)

Further, histological impact was observed in order to detect long term injury in cells or tissues on specific target organs. No deaths or remarkable signs of external toxicity were observed in all groups. However, several histopathological alterations were observed in test water treated groups compared to control groups. To confirm these observations, histological scoring system was carried out in order to obtain a quantitative assessment of renal damages (Table 2). Kidney sections from normal water treated control rats displayed normal renal tissue structure, complete renal tubular epithelial cells and no obvious pathological changes in glomerular or renal interstitium without any signs of degeneration and necrosis (Fig. 5a) All scores in this group was considered zero (score – 0). Morphological changes including loss of brush border in less than 25% of tubular cells, tubular epithelial degeneration and integrity of basal membrane (Fig. 5b) were clearly observed in the rats treated with test water sample (T1) for 60 days (score - 1). Light microscope evaluation of kidneys in 90 days of test water treated group (T2) associated with the acute tubular injury with loss of brush border in more than 25% of tubule cells and thickened basal membrane (Fig. 5c). Despite the presence of acute tubular injury, the cellular debris in the proximal tubules was prominent and both shedding of few viable and necrotic cells into the tubular lumen were seen (score - 2). Distilled water treated control groups displayed normal renal tissue morphology (Fig. 5d) and in rats treated with distilled water for 60 days (T3), kidney sections revealed the nearly preserved kidney structure and showed normal tubular epithelial cells with a better morphology (Fig. 5e) compared to the test water treated group for 60 days (T1) and with the control groups (score - 0). Moreover, distilled water treated group for 90 days (T4) showed minimal loss of brush border and appeared to be negligible. It was maintained a nearly normal morphology (Fig. 5f) compared with the 90 days of test water treated group (T2) and control groups (score - 0). Further, both, T3 and T4 groups showed near complete regeneration with intact nuclei and regular nuclei outline with eosinophilic cytoplasm.

**Table 2:** Renal histological scores and severity of tubular injury in rats treated with normal water, test water and distilled water for 60 and 90 days

	Groups		Score	Interpretation
Experiment I	60 days	N1	0	Normal
		T1	1	Acute tubular injury
	90 days	N2	0	Normal
		T2	2	Acute tubular injury
Experiment II	60 days	N3	0	Normal
		T3	0	Normal
	90 days	N4	0	Normal
		T4	0	Normal^a^

0. No damage

1 Loss of Brush Border (BB) in less than 25% of tubular cells. Integrity of basal membrane

2. Loss of BB in more than 25% of tubular cells, Thickened basal membrane

3. (Plus) Inflammation, Cast formation, Necrosis up to 60% of tubular cells

4. (Plus) Necrosis in more than 60% of tubular cells

0 = no tubular injury, 1–2 = acute tubular injury and 3–4 = chronic tubular injury; ^a^minimal loss of brush border and appeared to be negligible.

When compared the liver histology of the normal water, test water and distilled water treated rats, no significant histopathological changes or abnormalities were observed at all the time.

### Exposure to test water results in elevated serum creatinine and urea levels

**Fig. 6:**
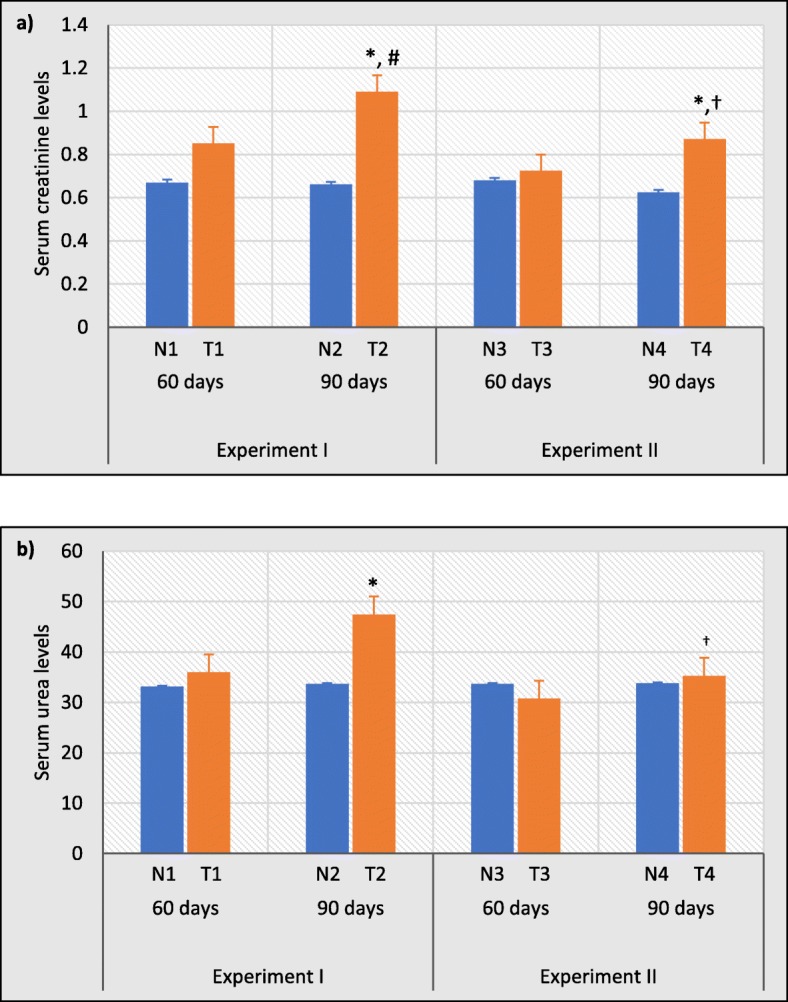
Effects of normal water and test water sample (experiment I) and distilled water treatment (experiment II) after 60 and 90 days of exposure on (**a**) serum creatinine levels and (**b**) serum urea levels as biochemical indicators of kidney function. ^*^p < 0.05 compared with respective control; ^#^*p* < 0.05 compared with T1; ^†^*p* < 0.05 compared with T2. Error bars represent standard error. Data are expressed as mean ± S.D., (n = 3)

Then the above histopathological results were compared with common renal biomarkers; serum creatinine and urea levels to estimate total renal function with respect to renal damage. Serum creatinine levels in experiment I were significantly increased by 39.45% (*p* < 0.05) after 90 days (T2) compared to respective control groups (Fig. 6a). Further, 90 days of test water treated group (T2) showed a significant increase by 22.02%; (*p* < 0.05) compared to test water treated group for 60 days (T1). In experiment II, both the creatinine levels in test groups have been decreased compared to experiment I. Moreover, the serum creatinine level in test group treated with distilled water for 90 days (T4) showed a significantly higher mean value (28.74%; *p* < 0.05) than its control group and a significant reduction compared to T2 in experiment I (25.29%; *p* < 0.05).

As similar to serum creatinine levels, serum urea levels in experiment I were also increased slightly in test water treated groups for 60 (T1) and significantly (28.98%; *p* < 0.05) at 90 (T2) days in comparison with their control groups treated with normal water while, slight decreased in distilled water treated test groups in experiment II compared to experiment I (Fig. 6b). However, the serum urea level of distilled water treated group for 90 days (T4) was significantly decreased (*p* < 0.05) compared to T2 group by 34.39%.

### AST activity was significantly increased after 90 days of test water administration

**Fig. 7:**
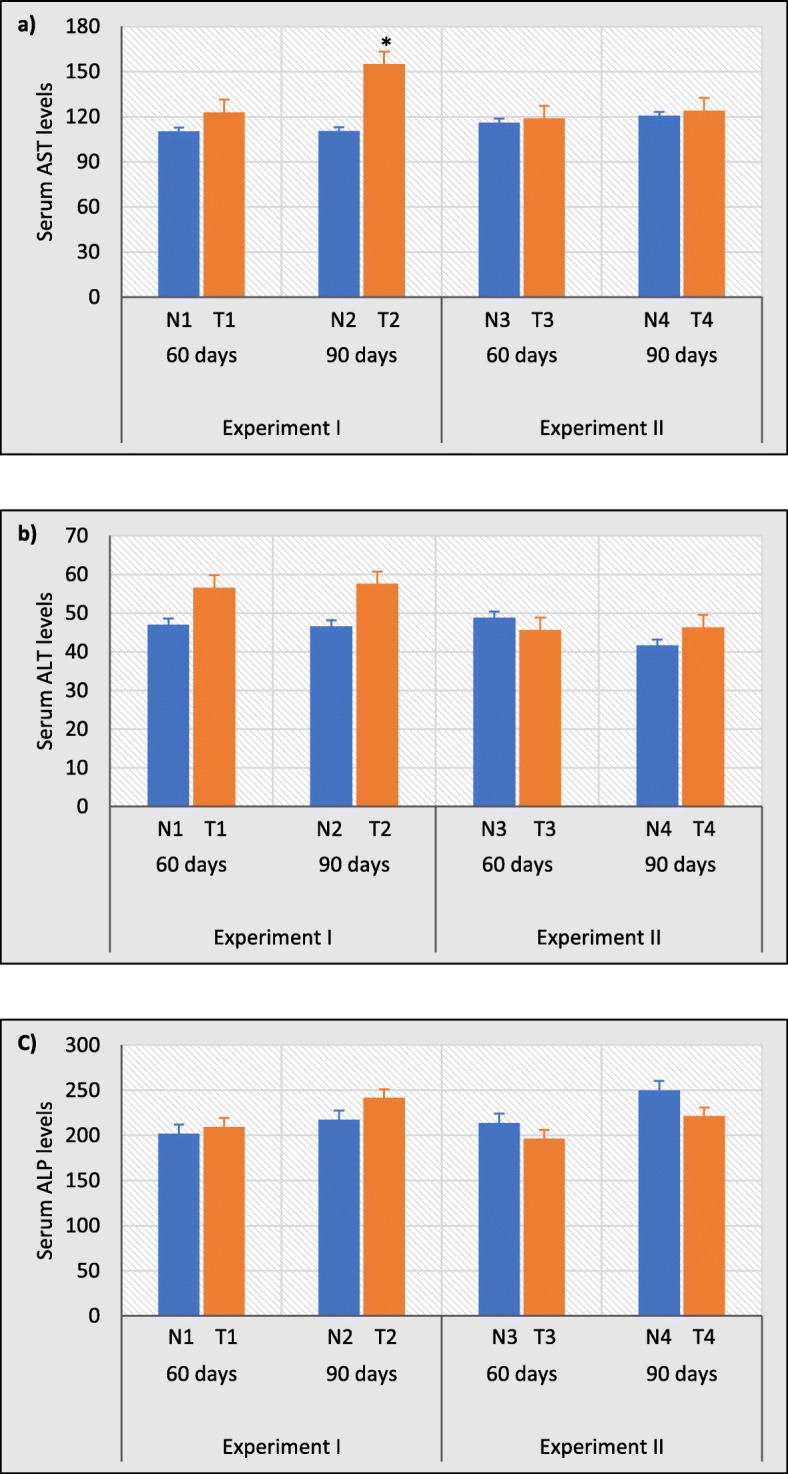
Effects of test water (experiment I) and distilled water treatment (experiment II) on serum (**a**) AST; aspartate aminotransferase, (**b**) ALT; alanine aminotransferase and (**c**) ALP; alkaline phosphatase as biochemical indicators of liver function. Error bars represent standard error. Data are expressed as mean ± S.D., (n = 3). *p < 0.05 compared with control

Although there were no liver histopathological changes, the increase or decrease in the activity of liver enzymes might indicate occurrence of liver injury. In this study, the levels of serum AST activity in experiment I was increased after administration of test water for 60 and 90 days when compared with their control groups and this increase was significant at 90 days by 28.41% (*p* < 0.05) compared to the respective control group (Fig. 7a). After providing distilled water in experiment II up to 60 and 90 days, the ALT activity was slightly decreased in test groups compared to experiment I, but the differences were not statistically significant.

Similarly, serum ALT and ALP activities were also showed increased levels in experiment I after 60 and 90 days compared to control groups and decreased levels of ALT and ALP activities after administration of distilled water in experiment II compared to experiment I. But these changes were statistically not significant (Fig. 7b and c).

### Serum electrolytes varied in the order Na^+^ > Ca^2+^ > Mg^2+^ > K^+^ and no significant differences were observed in fluoride levels

Serum Electrolytes level is a very good indicator of renal function and the balance of different electrolytes is vital for healthy function. The serum sodium levels were found to be much higher in experiment I and experiment II compared to the other electrolytes and the electrolytes varied in the order Na^+^ > Ca^2+^ > Mg^2+^ > K^+^ (Table 3). There was a significant reduction in serum sodium levels in T1 and T2 groups, potassium and magnesium levels in T1 groups compared to respective control groups. Further, serum calcium levels were significantly increase in T4 group compared to control group. The trace element concentrations in serum samples were extremely low or closed to the detection limits of the instrument and that will not be discussed further (data not shown).

**Table 3:** Effects of test water (experiment I) and distilled water treatment (experiment II) on serum electrolyte Na^+^, Ca^2+^, K^+^ and Mg^2+^levels. Data are expressed as mean ± S.D., (n = 3)

		Experiment I		Experiment II	
		60 Days	90 Days	60 Days	90 Days
Na	Control	42.484 ± 1.165	44.808 ± 1.495	30.816 ± 2.228	31.261 ± 4.487
	Test	34.408 ± 1.277^a^	35.972 ± 0.635^a^	32.276 ± 3.372	30.896 ± 2.710
Ca	Control	23.208 ± 1.416	26.385 ± 0.337	26.339 ± 0.686	21.811 ± 0862
	Test	25.109 ± 2.726	24.290 ± 2579	24.277 ± 1.003	24.947 ± 0.478^a^
K	Control	5.261 ± 0.282	4.779 ± 0.099	5.150 ± 0.534	5.001 ± 0.396
	Test	4.579 ± 0.320^a^	4.763 ± 0.323	5.282 ± 0.123	5.200 ± 0.309
Mg	Control	6.191 ± 0.638	6.877 ± 1.803	6.788 ± 1.110	4.655 ± 0.624
	Test	4.070 ± 0.875^a^	5.724 ± 1.836	7.374 ± 0.349	4.894 ± 0.576

^a^*p* < 0.05 compared with respective control

**Fig. 8:**
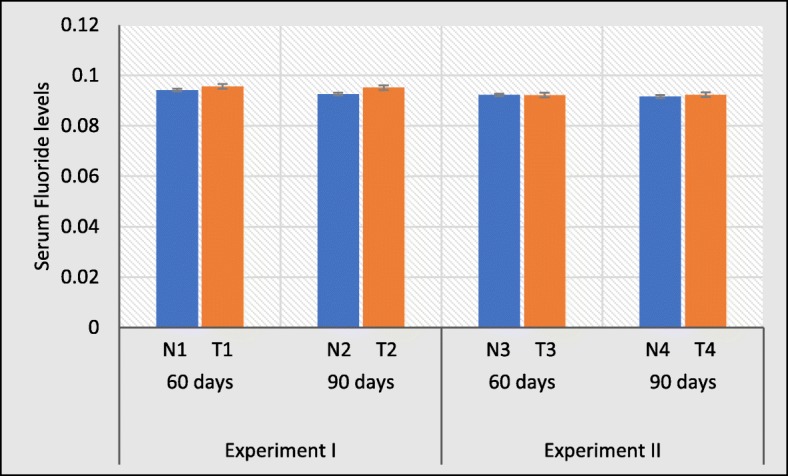
Effects of test water (experiment I) and distilled water treatment (experiment II) on serum fluoride levels. Error bars represent standard error. Data are expressed as mean ± S.D., (n = 3)

The estimation of fluoride in the serum is used to monitor the level of exposure as a good index of fluoride status in the human system [30]. The serum fluoride levels were not significantly higher in the test groups compared to control groups (Fig. 8). Similarly, no differences were observed for the serum level in distilled water treated groups.

## Discussion

Drinking water plays an important role for health and well-being. In our study, all the measured parameters were within the standard drinking water quality values of WHO, except pH, water alkalinity, hardness and fluoride levels. Drinking water must have a pH value of 6.5–8.5 to fall within standards. Drinking water with a low pH can be acidic and with a pH level above 8.5 indicates that the water is alkaline or hard and high levels of alkalinity minerals are present [31, 32]. Alkalinity is a measure of buffering capacity of water and it is an important parameter indicating ability of water to neutralize acid. Generally, it is associated with high pH values, hardness and excessive dissolved solids. Alkalinity is not considered detrimental to humans and appear to have no adverse health effects yet alkaline diets may result in a number of health benefits [33]. Moreover, such studies have reported that alkalinity is often related to hardness [34] and most of the time, this value is much closer to water hardness in CKDu regions [12]. Yet, there aren’t enough research to support the health claims made by alkalinity, we excluded the water pH and alkalinity parameters in this study. In this study, the total hardness in the test water sample was found to be 364 mg/l which was falling in the “very hard water” category. In a reason study, conducted by Wickramarathna et al (2017) has testified that the majority of the water samples from CKDu regions are plotted above the hard zone [12]. The content of fluoride in test water sample was 1.66 ppm and this value exceeded the 0.5 ppm, the limit recommended for tropical countries by WHO. Some studies revealed that other than fluoride, alterations in the other constituents that are present in the water such as Calcium (Ca), Sodium (Na), and possibly Magnesium (Mg) could be potential factors in the development of CKDu in the NCP [7]. Geochemically, water from the dry zone region is abundant in cations of Na^+^ and/or Ca^2+^. Furthermore, water hardness with high Ca concentrations compared to Na concentrations (Na/Ca) in drinking water have also said to be influencing CKDu [8]. According to our results, water sample collected from CKDu endemic area was characterized by low Na/Ca ratio of 2.673. Such studies have shown that sources of drinking water in affected and non-affected regions have different ratios of calcium and sodium despite having similar levels of fluoride. Chandrajith et al (2011) have reported that Na/Ca ratio in a range of 1.6 to 6.6 in the CKDu endemic areas as opposed to 34 and 469 in the non-endemic regions [8].

The body weights of rats administered with test water and the groups that received distilled water along with the control groups were increased, but the body weight gain difference was statistically not significant throughout the study compared to control groups. This was substantiated by an experiment conducted by Graf *et al* to investigate the effect of water hardness on lactating dairy cattle and no significant differences in body weight changes were observed in hard water consumed group (290 ppm) compared to control (0 ppm) group [35]. In our previous study, it has already shown that no indication of fluoride ingestion and significant body weight gain changes [22] and this was further supported by Tsunoda et al. [36] who have not seen any significant changes in body weights in fluoride treated groups up to 125 ppm. On the other hand, the experimental animals fed with NaF and calcium showed no changes in the weight loss [37]. However, organ weights of treated and untreated animals did not reveal a clear image of treatment related effects due to differences in body weights between the groups. Therefore, other parameters that are commonly used for analysis of organ weight are the ratio of the organ weight to body weight [38]. Reasons cited for the usefulness of weighing kidneys and liver in toxicity studies included: its sensitivity to predict toxicity, enzyme induction, physiologic perturbations and acute injury; it is frequently a target organ of toxicity; it correlates well with histopathological changes; and historical control range data is available [39]. According to the results, no statistically significant differences were found in relative kidney and liver weights of rats treated with test water sample for 60 and 90 days compared to control groups and distilled water treated groups. Further, these results have been confirmed with similar studies with different calcium levels [40] and fluoride levels [22]. Therefore, water hardness and fluoride together had no influence on body weight gain and organ weight changes.

Kidney is an important organ of the body that plays a principal role in homeostasis by excreting urine by filtering waste products from the blood stream [41]. Light microscopic evaluation of kidneys in 60 and 90 days of hard water treatment resulted with acute tubular injury (ATI). Acute tubular injury is renal failure that is the result of either ischemic or toxic insult to the kidney with evidence of damage to the tubules and the tubules appear to be a major site of injury in acute renal failure. The tubules are especially vulnerable for nephrotoxicity because active secretion and reabsorption of drugs and metabolites occur in these areas [42]. The factors which cause lesions in the renal tubular cells can lead to cell death or detachment from basement membrane causing tubular dysfunction [43]. According to the renal biopsy studies, at the initial stage of the cell injury, the proximal tubule shows apical blebs, loss of brush border in tubular segments, swelling of the cell cytoplasm, detachment of renal tubular epithelial cells from the basement membrane and sloughing of cells into the tubular lumen, etc. [43, 44]. The apical surface of the proximal tubule consists of the brush border, which is the specialized area for reabsorption and contains microvilli. Some studies have reported that the first lesion in the proximal epithelial cell is the loss of brush border [45]. Therefore, the disruption and loss of microvillar density are the hallmarks of renal tubular cell injury contributing to the development and progression of kidney diseases. Moreover, such studies have indicated that renal tubular damage occurs in the very early stage of CKDu [46]. When comparing these results with our observations, the early stages of ATI would probably not be much different. The major route of excretion of ingested fluoride from the body is the urine. Therefore, many studies have been designed to identify their effect on renal tissues as they are more susceptible to fluoride toxicity. Even though much high levels of fluoride are not consumed, experimental studies have shown a number of adverse health effects and a significant correlation between high fluoride levels (50 ppm – 250 ppm) and renal damage [36, 47, 48]. But studies with fairly low concentrations of fluoride were relatively limited and such studies have clearly revealed that consumption of water up to 8 ppm fluoride in drinking water had no evidence of an increased frequency of kidney disease or tubular dysfunction [49]. In relation to the CKDu in Sri Lanka, the recently published studies have shown that there were no remarkable changes in renal tissues with reported fluoride levels in drinking water at the concentrations of 0.5 ppm and 5 ppm [22]. Moreover, no histopathological changes were observed even at the highest concentrations tested (20 ppm). Therefore, fluoride alone has no risk of developing kidney disease. However, Na/Ca ratio in drinking water with high levels of Fluoride has been studied as a suspected cause of CKDu. When the Na^+^/Ca^2+^ ratio is high in water, fluoride combine with Na^+^ to form Sodium fluoride (NaF) which is soluble in water and reduces the toxicity of fluoride ions in the human body. On the other hand, higher Ca^2+^ levels which cause low Na^+^/Ca^2+^ ratios cause the damage on kidney tubular cells in the presence of fluoride by forming Calcium fluoride (CaF_2_), which is insoluble in water [8]. Calcium balance is tightly regulated by the concerted action of calcium absorption in the intestine, reabsorption in the kidney, and exchange from bones [50]. Calcium plays a critical role in many cell functions and it has a high affinity with fluoride ions and it’s binding with calcium causes ectopic calcification in teeth, bone, and soft tissues [51]. Some studies have shown that plasma levels of Ca were lower during the intake of sodium fluoride than in the control studies indicating decreased absorption of Ca [52]. Parathyroid hormone (PTH) is responsible for calcium homeostasis [53] and fluoride affects calcium homeostasis by regulating PTH, PTH-related peptide, and calcium-sensing receptor expression [54]. There can be many cellular factors that are involved with injury of the epithelial cell and some of them are ATP depletion, increased intracellular free Ca^2+^ concentration, reactive oxygen species, increased mitochondrial and plasma membrane permeability etc. [55]. Some studies have reported that fluoride increases intracellular calcium and causes renal calcification in rat renal epithelial cells [56]. Tubular cell calcium overload has been associated with altered function at the level of the plasma membrane, mitochondria, endoplasmic reticulum and cytoskeleton [57]. As ATP levels decrease, the intracellular sodium concentration ensuing Ca2+ to enter into the cell through the sodium-calcium exchanger by lowering the activity of the sodium-potassium ATPase pump on the plasma membrane [58]. Mitochondria are also actively involved in the maintenance of cellular Ca2+ homeostasis. But excessive Ca2 + overload may lead to mitochondrial damage, which ultimately result in cell injury and death [59]. Calcium ions may also activate enzymes that generate reactive oxygen or nitrogen species (ROS and/or RNS) [60] and impair ATP synthesis by causing oxidative injury to the inner membrane and also increases ATP consumption by the Ca^2+^-ATPases working to eliminate the excess Ca^2+^ [61]. Further, some studies have shown that high sodium intake induces intra-renal oxidative stress via increased NAD(P)H oxidase (NOX) activity and decreased expression of superoxide dismutase (SOD) [62]. And also increases the urinary elimination of calcium in proportion to sodium and water via reduced passive reabsorption of calcium [63]. As calcium transport is closely linked to sodium transport in renal tubules, high dietary sodium tends to decrease calcium reabsorption and increase calcium excretion via urine leading to a temporary decrease in serum calcium. This result in increase in parathyroid hormone [64].. Long term high-salt diet decreased claudin-2 and the reduction in claudin-2 protein expression may be partly responsible for the reduced Ca^2+^ reabsorption in the proximal tubules [65]. High calcium excretion is also related to the increasing bone resorption marker [66]. Further, some studies have shown that there is a positive relationship between water hardness and urinary stone incidence [67]. Although water hardness has been suggested as a causal factor that may contribute to the development of the disease in Sri Lanka, common characteristics across CKDu studies do not include urinary calculus as a risk factor [68]. No correlation was found in many studies between the urinary stone formation and the amount of calcium, bicarbonate, or the total hardness of the drinking water. Moreover, some experiments have shown that fluoride has a therapeutic value in prevention of renal stone formation. They have observed that fluoride can inhibit renal stone formation induced by Ethylene glycol by decreasing oxalate synthesis and urinary oxalate excretion [69]. However, the combined interaction of epidemiology, environmental exposure, dietary habits, and genetic factors may give rise to kidney stone disease [70, 71]. The promotion of higher intakes of calcium has also come under scrutiny because this will likely lead to high urinary calcium excretion (UCaE) and kidneys may be susceptible to damage from high UCaE [72].

Measurements of some biochemical markers can demonstrate harmful changes in kidney function, thereby serving as indices of renal function. Serum creatinine and urea are frequently measured as screening markers for renal dysfunction; thus, the increasing of serum levels of these markers are indicators of renal injury [73].

In this study, it was observed that the groups treated with hard water showed a considerable increase in serum creatinine and urea levels following 60 and 90 days of exposure to normal water and a reduction in serum creatinine and urea levels among those groups received distilled water as a treatment compared to hard water treated groups. Changes in serum creatinine between hard water and normal water treated rats suggest that this model of acute tubular injury is robust. Some studies have shown that calcium supplementation caused a slight increase in blood creatinine [74]. The mechanism by which calcium supplementation increases blood creatinine is unknown, but could be due to renal impairment. The morphological changes would contribute partially to disturbed tubular reabsorption and can explain why the serum urea and creatinine levels were significantly increased (*P* < 0.05) in hard water treated group as compared to their levels in the control groups. Mokhtar et al stated that the levels of serum urea and creatinine were more greatly increased in NaF and Ca treated group compared to the control and sodium fluoride alone treated groups [75].

The liver is the largest internal organ in the human body and it is responsible for the metabolism and detoxification of drugs and xenobiotics [60]. In our previous study, fluoride exposure resulted varies degrees of portal inflammation and focal necrosis on hepatic histology resulting in mild hepatic inflammation [22]. But in this experiment, qualitative assessment of tissue sections of the liver showed no damage in the experimental groups that received either normal water, hard water or distilled water. Other recent study has also been demonstrated that intake of calcium does not significantly related to the risk of mortality due to liver disease [76]. However, no study has investigated the association between direct intake of calcium and risk of liver damage. This was further confirmed by no significant differences in liver enzyme parameters, ALT and ALP activities. Such studies have shown that calcium supplementation associated with significant variances in microbial communities of intestine and feces. And also with decreased hepatic concentration of the primary conjugated murine bile acid (and hepatic farnesoid X receptor antagonist) taurine- β-MCA. These microbial and metabolic alterations may improve liver metabolic function [77]. Elevated serum AST activity can be indicative of impaired liver or renal function. AST is present in cytosolic and mitochondrial isoenzymes and is found in the liver, cardiac muscle skeletal muscle, kidneys, brain, pancreas, lungs, leucocytes, and red cells [78]. Therefore, it is less sensitive and specific for the liver. However, some studies have shown that AST and ALT serum levels tended to be higher during the initial stages (2 and 3) of CKD compared with the later stages (4 and 5) [79]. However, some studies have shown marked inhibitory effect on iron absorption of calcium [80, 81] which can accumulate in the liver. Therefore, calcium may indirectly affects the liver.

In our study, no significant differences were observed in serum electrolyte levels and trace element concentrations between hard water treated groups and distilled water treated groups compared to controls. According to Wijkstrom et al. [68], Electrolyte disturbances with low levels of serum sodium, potassium, and/or magnesium were common in CKDu patients in Sri Lanka [68]. This may be because of high renal output of electrolytes will make patients more susceptible to salt depletion and/or dehydration due to sweating in hot climates. We have previously reported that the rats treated with 5 mg/l F^−^ for 15, 30 and 60 days did not alter serum fluoride levels compared to control groups, although the dose of the fluoride was much higher than the doses used in the present study. However, in this study also showed a similar result for normal and test water treated groups for 60 and 90 days. This may be because most of the absorbed fluoride is taken up by mineralized tissues of the body such as; bone, enamel, and dentin, where fluoride is strongly but not irreversibly bound and it can be released back into the plasma according to the demand [82]. Further, fluoride absorption also dependent on dietary calcium levels and some studies have shown that fluoride ingested with high concentration of calcium may reduce fluoride absorption [83]. This was further confirmed by the results from animal experiments that chronically elevated plasma fluoride levels can be reduced by a diet rich in calcium resulting fluoride loss [82].

According to the results in experiment II, distilled water is effective in minimizing the histopathological and biochemical changes, but the correlative explanations and the actual mechanism is not known. However, renal tubules have a remarkable capacity to repair itself and regenerate lost cells following AKI, usually within less than a week [84]. The predominant recovery process is associated with the surviving tubular epithelial cells that remain adherent [55]. The viable cells spread, dedifferentiate, and migrate to cover the exposed areas of the basement membrane, and then proliferate to restore cell number. Replacement of the lost epithelial cells during repair by several mechanisms, ultimately recover functional integrity of the tubule [45, 55]. Furthermore, ingested distilled water add electrolytes by taking from the body reserves and also leads to the dilution of the electrolytes in the body. Also, some studies have presumed that intake of low-mineral water has facilitated the elimination of minerals from the body [85]. Therefore, there may be a correlation between elimination of electrolytes from the body and recovery of renal tubular damage. However, the real mechanisms cannot be invoked, though, some studies have shown its regular or large consumption can be considered as a potential health risk due to substantial lack of essential minerals in it.

Although, the findings in animal toxicology studies generally are applicable to humans, this study also provide certain limitations such as effects of human’s endogenous and exogenous factors like diet, stress, personal habits etc. and duration of an individual’s exposure to the chemical of interest in epidemiological studies and case histories. Further, small sample size can reduce the sensitivity of the study to detect adverse effects and too large sample size will also lead in a waste of scientific resources and animals [86]. Despite these limitations, data derived from this study clearly confirmed the clinical picture of CKDu in Sri Lanka.

## Conclusion

According to this study, high fluoride with hard water administration resulted acute tubular injury by destruction of renal tubules with a significantly increased serum levels of creatinine, urea and AST and these pathological changes were improved by introducing distilled water. Therefore, initial damage can occur even in the younger cohorts who drink water with high fluoride and hardness and it can be reversed if spotted in the early stages by introducing distilled or low solute containing pure water.

## Data Availability

The datasets analyzed during the current study are available from the corresponding author on reasonable request.

## Abbreviations

CKD: Chronic kidney disease
CKDu: Chronic kidney disease of unknown etiology
EC: Electrical conductivity
TDS: Total dissolved solids
F: Fluoride
K: Potassium
Na: Sodium
Ca: Calcium
Mg: Magnesium
Cu: Copper
Sr: Strontium
Mn: Manganese
Zn: Zinc
Ni: Nickel
Li: Lithium
Fe: Ferrous
HE: Haematoxylin and eosin
EGTI: Endothelial, Glomerular, Tubular, Interstitial
AST: Aspartate aminotransferase
ALT: Alanine aminotransferase
ALP: Alkaline phosphatase
ATI: Acute tubular injury
NaF: Sodium fluoride
CaF_2_: Calcium fluoride
PTH: Parathyroid hormone
